# 5-[(*E*)-(2-Fluoro­benzyl­idene)amino]-2-hy­droxy­benzoic acid

**DOI:** 10.1107/S160053681103162X

**Published:** 2011-08-17

**Authors:** M. Nawaz Tahir, Muhammad Ilyas Tariq, Riaz H. Tariq

**Affiliations:** a Department of Physics, University of Sargodha, Sargodha, Pakistan; b Department of Chemistry, University of Sargodha, Sargodha, Pakistan; c Institute of Chemical and Pharmaceutical Sciences, The University of Faisalabad, Faisalabad, Pakistan

## Abstract

In the title compound, C_14_H_10_FNO_3_, the dihedral angle between the two benzene rings is 32.66 (14)°. An *S*(6) ring motif is formed due to an intra­molecular O—H⋯O hydrogen bond between the hy­droxy and carbonyl groups. In the crystal, mol­ecules are consolidated into dimers with *R*_2_^2^(8) ring motifs by pairs of O—H⋯O hydrogen bonds.

## Related literature

For background and related crystal structures, see: Tahir & Shad (2010 ▶); Tahir *et al.* (2010*a* ▶,*b* ▶,*c* ▶). For graph-set notation, see: Bernstein *et al.* (1995 ▶).
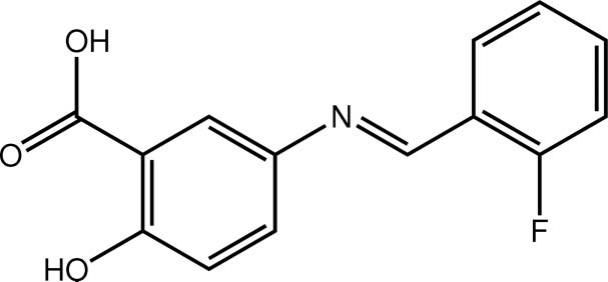


## Experimental

### 

#### Crystal data

C_14_H_10_FNO_3_
*M* *_r_* = 259.23
Monoclinic, 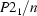

*a* = 15.5688 (16) Å
*b* = 4.7139 (4) Å
*c* = 16.2248 (16) Å
β = 92.412 (4)°
*V* = 1189.7 (2) Å^3^
*Z* = 4
Mo *K*α radiation
μ = 0.11 mm^−1^
*T* = 296 K
0.30 × 0.22 × 0.18 mm

#### Data collection

Bruker Kappa APEXII CCD diffractometer
Absorption correction: multi-scan (*SADABS*; Bruker, 2005 ▶) *T*_min_ = 0.972, *T*_max_ = 0.983
15880 measured reflections
2161 independent reflections
1156 reflections with *I* > 2σ(*I*)
*R*_int_ = 0.079

#### Refinement

*R*[*F* ^2^ > 2σ(*F* ^2^)] = 0.048
*wR*(*F* ^2^) = 0.127
*S* = 1.01
2161 reflections
179 parameters
H atoms treated by a mixture of independent and constrained refinement
Δρ_max_ = 0.17 e Å^−3^
Δρ_min_ = −0.16 e Å^−3^

### 

Data collection: *APEX2* (Bruker, 2009 ▶); cell refinement: *SAINT* (Bruker, 2009 ▶); data reduction: *SAINT*; program(s) used to solve structure: *SHELXS97* (Sheldrick, 2008 ▶); program(s) used to refine structure: *SHELXL97* (Sheldrick, 2008 ▶); molecular graphics: *ORTEP-3* (Farrugia, 1997 ▶) and *PLATON* (Spek, 2009 ▶); software used to prepare material for publication: *WinGX* (Farrugia, 1999 ▶) and *PLATON*.

## Supplementary Material

Crystal structure: contains datablock(s) global, I. DOI: 10.1107/S160053681103162X/tk2774sup1.cif

Structure factors: contains datablock(s) I. DOI: 10.1107/S160053681103162X/tk2774Isup2.hkl

Supplementary material file. DOI: 10.1107/S160053681103162X/tk2774Isup3.cml

Additional supplementary materials:  crystallographic information; 3D view; checkCIF report

## Figures and Tables

**Table 1:** Hydrogen-bond geometry (Å, °)

*D*—H⋯*A*	*D*—H	H⋯*A*	*D*⋯*A*	*D*—H⋯*A*
O1—H1⋯O2^i^	0.88 (3)	1.77 (3)	2.642 (3)	176 (3)
O3—H3⋯O2	0.92 (3)	1.78 (3)	2.617 (3)	152 (3)

Symmetry code: (i) 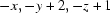.

**Fig. 1.**
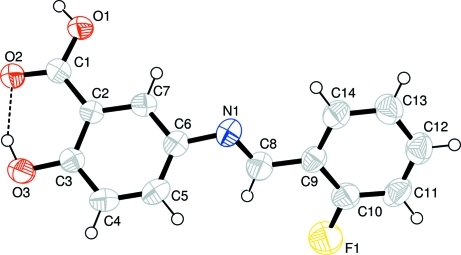
View of the title compound with the atom numbering scheme. The displacement ellipsoids are drawn at the 50% probability level. H-atoms are shown by small circles of arbitrary radii. The dotted line represents the intramolecular hydrogen bond.

**Fig. 2.**
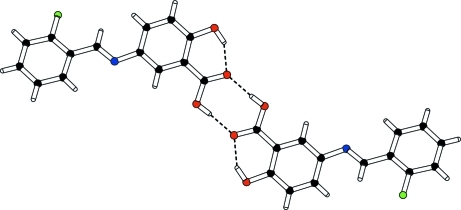
The partial packing showing that molecules form dimeric aggregates via O—H···O hydrogen bonds (dotted lines).

## supplementary crystallographic information

### Comment

Recently, we reported crystal structures containing the 5-amino-2-hydroxybenzoic acid moiety *i.e.* (II) 2-hydroxy-5-[(*E*)-(1*H*-indol-3-ylmethylidene)azaniumyl]benzoate (Tahir & Shad, 2010), (III) *i.e.* 2-{[(*E*)-1,3-benzodioxol-5-yl]methylideneamino}benzoic acid (Tahir *et al.*, 2010*b*), (IV) *i.e.* 5-[(*E*)-(2,6-dichlorobenzylidene)amino]-2-hydroxybenzoic acid (Tahir *et al.*, 2010*a*) and (V) *i.e.* 2-hydroxy-5-{[(*E*)-4-methoxybenzylidene]azaniumyl}benzoate (Tahir *et al.*, 2010*c*). The title compound (I), (Fig. 1) was prepared in continuation of the synthesis of various molecules having 5-amino-2-hydroxybenzoic acid.

In (I), group A (C1—C7/N1/O1—O3), derived from 5-amino-2-hydroxybenzoic acid, and group B (C8—C14/F1), derived from 2-fluorobenzaldehyde, are each planar with r.m.s. deviations of 0.0164 and 0.0182 Å, respectively. The A/B dihedral angle between is 32.78 (7)°. There exists an intramolecular O—H···O hydrogen bond which completes a S(6) ring motif (Table 1, Fig. 1). In the crystal packing the molecules are stabilized in the form of dimers due to intermolecular O—H···O hydrogen bonds (Table 1, Fig. 2) which form a *R*_2_^2^(8) ring motif (Bernstein *et al.*, 1995).

### Experimental

Equimolar quantities of 5-amino-2-hydroxybenzoic acid and 2-fluorobenzaldehyde were refluxed in methanol for 45 min resulting in yellow-brown solution. The solution was kept at room temperature which afforded violet prisms after 48 h.

### Refinement

The coordinates of the hydroxyl-H atoms were refined freely, and with *U*_iso_(H) = 1.2*U*_eq_(O). The C-bound H-atoms were positioned geometrically (C–H = 0.93 Å) and refined as riding with *U*_iso_(H) = 1.2*U*_eq_(C).

### Crystal data

**Table:**

C_14_H_10_FNO_3_	*F*(000) = 536
*M**_r_* = 259.23	*D*_x_ = 1.447 Mg m^−^^3^
Monoclinic, *P*2_1_/*n*	Mo *K*α radiation, λ = 0.71073 Å
Hall symbol: -P 2yn	Cell parameters from 1156 reflections
*a* = 15.5688 (16) Å	θ = 2.5–25.3°
*b* = 4.7139 (4) Å	µ = 0.11 mm^−^^1^
*c* = 16.2248 (16) Å	*T* = 296 K
β = 92.412 (4)°	Prism, violet
*V* = 1189.7 (2) Å^3^	0.30 × 0.22 × 0.18 mm
*Z* = 4	

### Data collection

**Table:**

Bruker Kappa APEXII CCD diffractometer	2161 independent reflections
Radiation source: fine-focus sealed tube	1156 reflections with *I* > 2σ(*I*)
graphite	*R*_int_ = 0.079
Detector resolution: 8.10 pixels mm^-1^	θ_max_ = 25.3°, θ_min_ = 2.5°
ω scans	*h* = −18→18
Absorption correction: multi-scan (*SADABS*; Bruker, 2005)	*k* = −5→5
*T*_min_ = 0.972, *T*_max_ = 0.983	*l* = −19→19
15880 measured reflections	

### Refinement

**Table:**

Refinement on *F*^2^	Secondary atom site location: difference Fourier map
Least-squares matrix: full	Hydrogen site location: inferred from neighbouring sites
*R*[*F*^2^ > 2σ(*F*^2^)] = 0.048	H atoms treated by a mixture of independent and constrained refinement
*wR*(*F*^2^) = 0.127	*w* = 1/[σ^2^(*F*_o_^2^) + (0.0531*P*)^2^ + 0.0687*P*] where *P* = (*F*_o_^2^ + 2*F*_c_^2^)/3
*S* = 1.01	(Δ/σ)_max_ < 0.001
2161 reflections	Δρ_max_ = 0.17 e Å^−^^3^
179 parameters	Δρ_min_ = −0.16 e Å^−^^3^
0 restraints	Extinction correction: *SHELXL97* (Sheldrick, 2008), Fc^*^=kFc[1+0.001xFc^2^λ^3^/sin(2θ)]^-1/4^
Primary atom site location: structure-invariant direct methods	Extinction coefficient: 0.012 (2)

### Special details

**Table:**

Geometry. Bond distances, angles *etc*. have been calculated using the rounded fractional coordinates. All su's are estimated from the variances of the (full) variance-covariance matrix. The cell e.s.d.'s are taken into account in the estimation of distances, angles and torsion angles
Refinement. Refinement of *F*^2^ against ALL reflections. The weighted *R*-factor *wR* and goodness of fit *S* are based on *F*^2^, conventional *R*-factors *R* are based on *F*, with *F* set to zero for negative *F*^2^. The threshold expression of *F*^2^ > σ(*F*^2^) is used only for calculating *R*-factors(gt) *etc*. and is not relevant to the choice of reflections for refinement. *R*-factors based on *F*^2^ are statistically about twice as large as those based on *F*, and *R*- factors based on ALL data will be even larger.

### Fractional atomic coordinates and isotropic or equivalent isotropic displacement parameters (Å^2^)

**Table:**

	*x*	*y*	*z*	*U*_iso_*/*U*_eq_	
F1	0.52929 (12)	−0.3124 (5)	0.56845 (13)	0.1012 (9)	
O1	0.06399 (12)	0.7307 (4)	0.55258 (12)	0.0525 (8)	
O2	0.05067 (11)	0.8698 (4)	0.42115 (11)	0.0495 (7)	
O3	0.14217 (13)	0.6175 (4)	0.31108 (12)	0.0542 (8)	
N1	0.28499 (15)	−0.0194 (4)	0.57191 (14)	0.0515 (9)	
C1	0.08505 (17)	0.7167 (5)	0.47509 (18)	0.0417 (10)	
C2	0.15088 (16)	0.5080 (5)	0.45759 (15)	0.0375 (9)	
C3	0.17635 (17)	0.4699 (5)	0.37661 (17)	0.0434 (10)	
C4	0.23883 (18)	0.2707 (6)	0.36048 (19)	0.0519 (11)	
C5	0.27501 (17)	0.1124 (6)	0.42340 (19)	0.0514 (11)	
C6	0.25157 (17)	0.1477 (5)	0.50467 (18)	0.0442 (10)	
C7	0.18911 (17)	0.3435 (5)	0.52047 (17)	0.0432 (9)	
C8	0.3618 (2)	−0.1094 (6)	0.57106 (19)	0.0549 (11)	
C9	0.39800 (18)	−0.2991 (5)	0.63403 (18)	0.0487 (11)	
C10	0.4808 (2)	−0.4013 (6)	0.6308 (2)	0.0584 (11)	
C11	0.5166 (2)	−0.5910 (7)	0.6868 (2)	0.0656 (12)	
C12	0.4681 (2)	−0.6790 (7)	0.7506 (2)	0.0651 (12)	
C13	0.3861 (2)	−0.5771 (7)	0.7575 (2)	0.0671 (12)	
C14	0.35127 (19)	−0.3924 (6)	0.70003 (19)	0.0607 (11)	
H1	0.0276 (18)	0.869 (6)	0.5597 (16)	0.0630*	
H3	0.1029 (18)	0.734 (6)	0.3343 (18)	0.0651*	
H4	0.25617	0.24473	0.30685	0.0624*	
H5	0.31634	−0.02248	0.41163	0.0618*	
H7	0.17190	0.36688	0.57425	0.0518*	
H8	0.39610	−0.05202	0.52858	0.0659*	
H11	0.57227	−0.65780	0.68150	0.0788*	
H12	0.49076	−0.80744	0.78922	0.0777*	
H13	0.35381	−0.63410	0.80153	0.0803*	
H14	0.29529	−0.32804	0.70528	0.0729*	

### Atomic displacement parameters (Å^2^)

**Table:**

	*U*^11^	*U*^22^	*U*^33^	*U*^12^	*U*^13^	*U*^23^
F1	0.0551 (12)	0.1469 (19)	0.1031 (17)	0.0089 (12)	0.0211 (12)	0.0358 (14)
O1	0.0594 (14)	0.0547 (12)	0.0440 (13)	0.0135 (10)	0.0101 (10)	−0.0004 (10)
O2	0.0534 (13)	0.0504 (12)	0.0450 (12)	0.0078 (10)	0.0043 (10)	0.0038 (10)
O3	0.0612 (14)	0.0565 (13)	0.0456 (13)	0.0044 (10)	0.0096 (10)	0.0015 (10)
N1	0.0474 (16)	0.0455 (14)	0.0613 (17)	0.0035 (12)	−0.0011 (13)	0.0003 (12)
C1	0.0439 (18)	0.0368 (15)	0.0443 (18)	−0.0081 (14)	0.0023 (15)	−0.0009 (14)
C2	0.0398 (16)	0.0336 (14)	0.0391 (17)	−0.0055 (12)	0.0034 (13)	−0.0014 (13)
C3	0.0431 (17)	0.0398 (15)	0.0476 (18)	−0.0057 (14)	0.0050 (14)	0.0029 (14)
C4	0.053 (2)	0.0519 (18)	0.0517 (19)	−0.0005 (16)	0.0132 (16)	−0.0083 (16)
C5	0.0437 (18)	0.0448 (17)	0.066 (2)	0.0021 (14)	0.0058 (16)	−0.0085 (16)
C6	0.0419 (17)	0.0376 (15)	0.0529 (19)	−0.0046 (14)	0.0009 (14)	−0.0005 (14)
C7	0.0454 (17)	0.0377 (15)	0.0469 (17)	−0.0032 (13)	0.0075 (14)	−0.0038 (13)
C8	0.049 (2)	0.0498 (17)	0.066 (2)	−0.0056 (15)	0.0025 (16)	0.0062 (16)
C9	0.0419 (18)	0.0461 (17)	0.058 (2)	0.0001 (14)	0.0006 (15)	−0.0005 (15)
C10	0.0444 (19)	0.068 (2)	0.063 (2)	−0.0011 (17)	0.0061 (17)	0.0017 (18)
C11	0.049 (2)	0.078 (2)	0.069 (2)	0.0194 (18)	−0.0075 (18)	−0.0099 (19)
C12	0.065 (2)	0.068 (2)	0.061 (2)	0.0096 (18)	−0.0120 (19)	0.0010 (17)
C13	0.056 (2)	0.085 (2)	0.060 (2)	0.0020 (19)	−0.0019 (17)	0.0117 (19)
C14	0.0460 (19)	0.069 (2)	0.067 (2)	0.0058 (17)	0.0003 (17)	0.0043 (18)

### Geometric parameters (Å, °)

**Table:**

F1—C10	1.354 (4)	C8—C9	1.454 (4)
O1—C1	1.314 (3)	C9—C14	1.391 (4)
O2—C1	1.239 (3)	C9—C10	1.379 (4)
O3—C3	1.360 (3)	C10—C11	1.376 (4)
O1—H1	0.88 (3)	C11—C12	1.371 (5)
O3—H3	0.92 (3)	C12—C13	1.373 (4)
N1—C6	1.426 (3)	C13—C14	1.371 (4)
N1—C8	1.270 (4)	C4—H4	0.9300
C1—C2	1.457 (4)	C5—H5	0.9300
C2—C7	1.395 (4)	C7—H7	0.9300
C2—C3	1.400 (4)	C8—H8	0.9300
C3—C4	1.385 (4)	C11—H11	0.9300
C4—C5	1.367 (4)	C12—H12	0.9300
C5—C6	1.393 (4)	C13—H13	0.9300
C6—C7	1.373 (4)	C14—H14	0.9300
			
C1—O1—H1	110.7 (17)	F1—C10—C9	118.1 (3)
C3—O3—H3	103.4 (18)	F1—C10—C11	118.1 (3)
C6—N1—C8	119.3 (2)	C10—C11—C12	118.3 (3)
O1—C1—O2	121.9 (2)	C11—C12—C13	120.0 (3)
O1—C1—C2	115.2 (2)	C12—C13—C14	120.6 (3)
O2—C1—C2	122.8 (3)	C9—C14—C13	121.3 (3)
C1—C2—C3	119.9 (2)	C3—C4—H4	120.00
C3—C2—C7	119.1 (2)	C5—C4—H4	120.00
C1—C2—C7	121.0 (2)	C4—C5—H5	119.00
O3—C3—C4	117.0 (2)	C6—C5—H5	119.00
O3—C3—C2	123.4 (2)	C2—C7—H7	119.00
C2—C3—C4	119.6 (2)	C6—C7—H7	119.00
C3—C4—C5	120.0 (3)	N1—C8—H8	119.00
C4—C5—C6	121.7 (3)	C9—C8—H8	119.00
C5—C6—C7	118.2 (3)	C10—C11—H11	121.00
N1—C6—C7	117.8 (2)	C12—C11—H11	121.00
N1—C6—C5	123.8 (2)	C11—C12—H12	120.00
C2—C7—C6	121.4 (3)	C13—C12—H12	120.00
N1—C8—C9	122.4 (3)	C12—C13—H13	120.00
C10—C9—C14	116.0 (3)	C14—C13—H13	120.00
C8—C9—C10	121.6 (3)	C9—C14—H14	119.00
C8—C9—C14	122.4 (3)	C13—C14—H14	119.00
C9—C10—C11	123.7 (3)		
			
C8—N1—C6—C5	33.0 (4)	C4—C5—C6—C7	1.4 (4)
C8—N1—C6—C7	−150.8 (3)	N1—C6—C7—C2	−177.6 (2)
C6—N1—C8—C9	−175.0 (2)	C5—C6—C7—C2	−1.3 (4)
O1—C1—C2—C3	178.2 (2)	N1—C8—C9—C10	178.1 (3)
O1—C1—C2—C7	−1.6 (3)	N1—C8—C9—C14	−0.8 (4)
O2—C1—C2—C3	−1.4 (4)	C8—C9—C10—F1	2.2 (4)
O2—C1—C2—C7	178.8 (2)	C8—C9—C10—C11	−177.0 (3)
C1—C2—C3—O3	−0.6 (4)	C14—C9—C10—F1	−178.9 (3)
C1—C2—C3—C4	−179.9 (2)	C14—C9—C10—C11	1.9 (4)
C7—C2—C3—O3	179.2 (2)	C8—C9—C14—C13	178.3 (3)
C7—C2—C3—C4	−0.1 (4)	C10—C9—C14—C13	−0.6 (4)
C1—C2—C7—C6	−179.6 (2)	F1—C10—C11—C12	179.2 (3)
C3—C2—C7—C6	0.6 (4)	C9—C10—C11—C12	−1.6 (5)
O3—C3—C4—C5	−179.1 (2)	C10—C11—C12—C13	−0.2 (5)
C2—C3—C4—C5	0.2 (4)	C11—C12—C13—C14	1.5 (5)
C3—C4—C5—C6	−0.8 (4)	C12—C13—C14—C9	−1.1 (5)
C4—C5—C6—N1	177.5 (3)		

### Hydrogen-bond geometry (Å, °)

**Table:**

*D*—H···*A*	*D*—H	H···*A*	*D*···*A*	*D*—H···*A*
O1—H1···O2^i^	0.88 (3)	1.77 (3)	2.642 (3)	176 (3)
O3—H3···O2	0.92 (3)	1.78 (3)	2.617 (3)	152 (3)

Symmetry codes: (i) −*x*, −*y*+2, −*z*+1.

## References

[bb1] Bernstein, J., Davis, R. E., Shimoni, L. & Chang, N.-L. (1995). *Angew. Chem. Int.* Ed. Engl. **34**, 1555-1573.

[bb2] Bruker (2005). *SADABS* Bruker AXS Inc., Madison, Wisconsin, USA.

[bb3] Bruker (2009). *APEX2* and *SAINT* Bruker AXS Inc., Madison, Wisconsin, USA.

[bb4] Farrugia, L. J. (1997). *J. Appl. Cryst.* **30**, 565.

[bb5] Farrugia, L. J. (1999). *J. Appl. Cryst.* **32**, 837–838.

[bb6] Sheldrick, G. M. (2008). *Acta Cryst.* A**64**, 112–122. 10.1107/S0108767307043930 18156677

[bb7] Spek, A. L. (2009). *Acta Cryst.* D**65**, 148–155. 10.1107/S090744490804362X PMC2631630 19171970

[bb8] Tahir, M. N. & Shad, H. A. (2010). *Acta Cryst.* E**66**, o3314. 10.1107/S1600536810048579 PMC3011759 21589591

[bb9] Tahir, M. N., Shad, H. A., Khan, M. N. & Tariq, M. I. (2010*a*). *Acta Cryst.* E**66**, o2672. 10.1107/S1600536810038420 PMC2983421 21587642

[bb10] Tahir, M. N., Shad, H. A., Khan, M. N. & Tariq, M. I. (2010*b*). *Acta Cryst.* E**66**, o2923. 10.1107/S160053681004136X PMC3009312 21589097

[bb11] Tahir, M. N., Tariq, M. I., Ahmad, S. & Sarfraz, M. (2010*c*). *Acta Cryst.* E**66**, o2553–o2554. 10.1107/S1600536810036172 PMC2983260 21587541

